# The Relationship Between Microbiomes and Selective Regimes in the Sponge Genus *Ircinia*

**DOI:** 10.3389/fmicb.2021.607289

**Published:** 2021-03-11

**Authors:** Joseph B. Kelly, David E. Carlson, Jun Siong Low, Tyler Rice, Robert W. Thacker

**Affiliations:** ^1^Department of Ecology and Evolution, Stony Brook University, Stony Brook, NY, United States; ^2^Limnological Institute University Konstanz, Aquatic Ecology and Evolution, Konstanz, Germany; ^3^Institute for Research in Biomedicine, Università della Svizzera Italiana, Bellinzona, Switzerland; ^4^Department of Immunobiology, Yale University School of Medicine, New Haven, CT, United States; ^5^Smithsonian Tropical Research Institute, Balboa, Panama

**Keywords:** microbiome, sponge (Porifera), 16S metabarcoding, RADseq, innate immunity

## Abstract

Sponges are often densely populated by microbes that benefit their hosts through nutrition and bioactive secondary metabolites; however, sponges must simultaneously contend with the toxicity of microbes and thwart microbial overgrowth. Despite these fundamental tenets of sponge biology, the patterns of selection in the host sponges’ genomes that underlie tolerance and control of their microbiomes are still poorly understood. To elucidate these patterns of selection, we performed a population genetic analysis on multiple species of *Ircinia* from Belize, Florida, and Panama using an *F*_*ST*_-outlier approach on transcriptome-annotated RADseq loci. As part of the analysis, we delimited species boundaries among seven growth forms of *Ircinia*. Our analyses identified balancing selection in immunity genes that have implications for the hosts’ tolerance of high densities of microbes. Additionally, our results support the hypothesis that each of the seven growth forms constitutes a distinct *Ircinia* species that is characterized by a unique microbiome. These results illuminate the evolutionary pathways that promote stable associations between host sponges and their microbiomes, and that potentially facilitate ecological divergence among *Ircinia* species.

## Introduction

Microorganisms affect nearly every aspect of macro-organismal biology. Across eukaryotes, the influences of microbiomes on hosts can be found in biological processes such as nutrition, development, and disease resistance (Freeman and Thacker, 2011; Berendsen et al., 2012; Clemente et al., 2012; Haney et al., 2015). These effects can be advantageous, for example, by producing essential nutrients for the hosts (Moran and Baumann, 2000) and by enabling the exploitation of novel resources (Falkowski et al., 1984; Long, 1989; Yeoman and White, 2014). An important characteristic of metazoan microbiomes concerns their stability, or at the very least the prevention of their overgrowth of the host, as commensal microbes can transition into opportunistic pathogens (Brown et al., 2012). The universal challenge of maintaining healthy associations with microbiomes is met by diverse strategies that include embargoes (Kiers et al., 2003), phagocytosis (Aderem and Underhill, 1999), and physical expulsions of microbes (Nyholm and McFall-Ngai, 2004). Beneath the many mechanisms of microbiome control might lie a common process among metazoans that polices the crosstalk between hosts and their microbes: the innate immune system. This proposition is supported by observations that pathways concerned with innate immune surveillance, especially in lipopolysaccharide (LPS) sensing, are functionally conserved across distantly related host clades. LPS is a common membrane-motif found in nearly all gram-negative bacteria and has been coopted as a prototypical endotoxin that binds to toll-like receptors (TLRs), instigating an immune response. Similarities have been documented between human and sponge LPS-induced pathways, including the likely role of toll-like receptors (TLRs) as pattern recognition receptors (PRRs) that bind LPS (Wiens et al., 2007). Furthermore, nodal signaling molecules downstream of the LPS-induced pathway in humans, serine-threonine-directed mitogen-activated protein kinases (MAPK) p38 kinases and c-*jun* N-terminal kinases/JNK (Sweet and Hume, 1996), are also stimulated by LPS in sponges (Böhm et al., 2001), a clade that is among the most distant metazoan relatives of humans. Given the homology between the components of the LPS-induced pathway and the fact that sponges and humans share a common ancestor at the base of the metazoan phylogeny (Dunn et al., 2015), these pathways might constitute extant versions of the innate immune system of ancient metazoans that facilitated inhabitation of hosts by symbiotic microbes in Earth’s early oceans, and which promote stable associations between hosts and microbes today.

Sponges stand out as a holobiont success story. The relationship between sponges and their microbes is a longstanding affair dating back to the advent of the phylum Porifera over 540 million years ago (Brunton and Dixon, 1994). Symbiotic microbes can comprise a substantial physical portion of sponge bodies, constituting up to 40% of the total biomass in some host species (Vacelet and Donadey, 1977; Wilkinson, 1978; Hentschel et al., 2006). Much like the zooxanthellae of coral, the cyanobacterial photosymbionts of sponges can supplement the host’s nutrition (Freeman and Thacker, 2011); although in some sponges that possess high abundances of symbiotic microbes, often termed high microbial abundance (HMA) sponges, cyanobacteria only constitute a portion of the microbial diversity (Thomas et al., 2016). The remaining fraction of these microbiomes can be composed of thousands of microbial species from dozens of bacterial phyla (Thomas et al., 2016) that perform fermentation (Hentschel et al., 2006), produce secondary metabolites (Thomas et al., 2010), conduct chemoautotrophic processes such as nitrogen fixation and sulfur oxidation (Tian et al., 2014; Archer et al., 2017; Slaby et al., 2017) and heterotrophic processes via the assimilation of dissolved organic matter (DOM) (Rix et al., 2020). Based on measurements of nutrient transfer from microbes to their hosts (Achlatis et al., 2018), supplements of microbial origin to the sponges’ energy pools that the hosts use for growth (Erwin and Thacker, 2008; Freeman and Thacker, 2011), and chemical defense by the secondary metabolites (Wilson et al., 2014; Lackner et al., 2017), microbial symbionts can influence the fitness of their hosts and shape their ecological identities.

The importance of microbiomes to HMA sponges is demonstrated by compositional stability and distinctness relative to the microbial communities of the surrounding environment (Thomas et al., 2016; Moitinho-Silva et al., 2017). The microbiomes can also be divergent among host species (Thomas et al., 2016) which, combined with the metabolic diversity of the microbes, supports the hypothesis that the microbiome acts as a mechanism for ecological diversification within sponges. This evolutionary model has received some of its strongest support in recent work investigating the bulk isotopic enrichment levels of sympatric sponge species (Freeman et al., 2020), where both microbiome compositions and isotopic enrichment values were divergent among host taxa within geographic sites, identifying the microbiomes as not only a mechanism for accessing novel resources but also a means for alleviating resource competition. Thus, microbiomes might also enable ecological diversification among incipient sponge species.

A suitable case study to test this hypothesis exists in *Ircinia*, a cosmopolitan sponge genus comprised of over 80 described species, many of which are densely populated by taxonomically diverse communities of symbiotic microbes (Erwin et al., 2012; Marino et al., 2017; Griffiths et al., 2019; van Soest et al., 2019). The microbiomes of several *Ircinia* are compositionally stable (Erwin et al., 2012; Pita et al., 2013) and unique among host species (Thomas et al., 2016). These microbes may supplement host nutrition given the high rates of primary productivity observed in *Ircinia* (Wilkinson and Cheshire, 1990) and isotopic evidence demonstrating the allocation of microbial nitrogen to the hosts (Weisz et al., 2007). Thus, the present study sought to use Caribbean *Ircinia* to investigate how patterns of selection in the hosts’ genomes promote the residence of symbiotic microbes which could, in turn, mediate ecological divergence among closely related host species. To perform this study, we first tested whether active control of microbiomes by the hosts is evidenced throughout *Ircinia* by characterizing beta diversity among the microbial consortia of the hosts and surrounding seawater using 16S rRNA metabarcoding. Second, we tested whether this control translates to dissimilar microbiome compositions among *Ircinia* host species, which we delimited independently using 2bRAD (RADseq) data, as the possession of unique microbiomes is congruent with prior evidence of ecological diversification within sponges (Freeman et al., 2020). Third, we investigated whether microbiomes are involved in ecological divergence among Caribbean *Ircinia* species by testing the hypothesis that genetic distance among the hosts correlates with microbiome dissimilarity. Finally, we identified *F*_*ST*_-outliers in the 2bRAD data and annotated them using a *de novo*-assembled and annotated transcriptome to find candidate genes that might underly divergence in microbiome control and genes that could facilitate the tolerance of symbionts.

## Materials and Methods

### Specimen Collections

**FIGURE 1:**
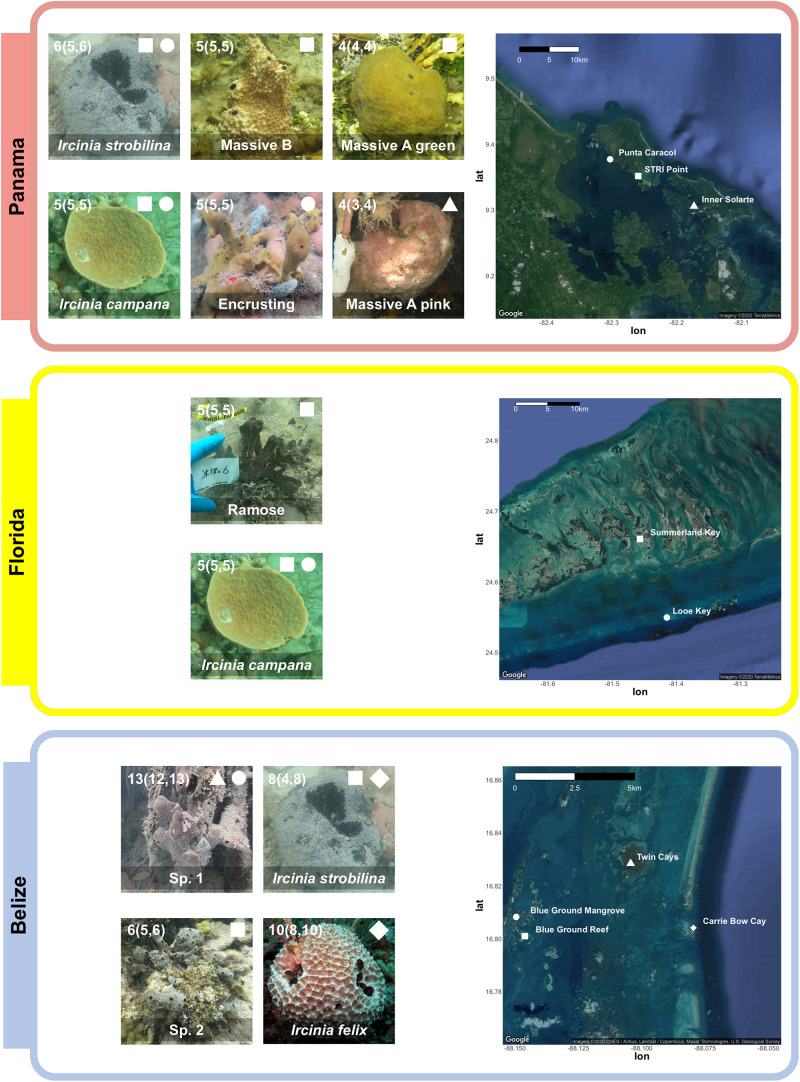
**Top**: Four Panamanian growth forms were collected from three sites: Massive A pink was collected from *Rhizophora* prop roots at Inner Solarte, a network of mangrove hammocks; Massive A green and Massive B were collected from STRI Point, a *Thalassia* seagrass-dominated habitat; and Encrusting was collected from Punta Caracol, a coral patch reef. *I. campana* and *I. strobilina* specimens were also collected from STRI Point and Punta Caracol. **Middle**: specimens of a growth form (Ramose) and *I. campana* were collected from a seagrass bed on Summerland Key, Florida; two specimens of *I. campana* were also collected from Looe Key. **Bottom**: Two Belizean growth forms were collected from three sites: Sp. 1 specimens were collected from *Rhizophora* prop roots at the Twin Cays and from mangrove hammocks adjacent to the series of Blue Ground coral patch reefs; and Sp. 2 specimens were collected from the coral reefs at Blue Ground. Specimens of *I. strobilina* were collected form the same patch reef inhabited by Sp. 2 and also from the forereef at Carrie Bow Cay, and *I. felix* specimens were collected from the Carrie Bow Cay forereef. *I. felix* photo credit: Patrick Erwin (Hentschel et al., 2012). Numbers in each photograph correspond to sample size, reported as total sample size (samples in 2bRAD dataset, samples in 16S dataset), less the transcriptomic sample of the Ramose growth form. A complete sampling overview can be found in Supplementary Table 1.

Specimens of *Ircinia* representing seven growth forms were collected from three sites: Bocas del Toro, Panama (July 2016), the Florida Keys (July 2018), and the Mesoamerican Barrier Reef (August 2018) (Figure 1 and Supplementary Table 1). Specimens of *I. campana* Lamarck 1814, *I. strobilina* Lamarck 1816, and *I. felix* Duchassaing and Michelotti 1864 were identified by comparing their internal and external morphology to previous species descriptions (van Soest, 1978). Morphological characteristics included microanatomical features, such as fiber widths and coring, as well as macroscopic features, such as conule height, tissue coloration, oscula size and orientation, and dermal reticulation. In Panama, individuals of the growth form Massive A pink were collected form mangrove prop roots of *Rhizophora* at Inner Solarte; individuals of two growth forms, Massive A green and Massive B, were collected from the seagrass-dominated (*Thalassia*) habitat of STRI point; and individuals of the Encrusting growth form were collected from patch reefs of Punta Caracol. In Florida, specimens of *I. campana* and a growth form with a branching body morphology (Ramose) were collected from a 150 m-long seagrass bed that begins 50 m immediately to the west of MOTE Marine Laboratory and Aquarium’s Elizabeth Moore International Center for Coral Reef Research & Restoration; *I. campana* specimens were also collected from the coral reef at Looe Key. An additional specimen of the Ramose growth form that was not included in either the 16S or 2bRAD dataset was snap frozen in an EtOH-dry ice bath and stored at -80C for subsequent RNA extraction. In Belize, specimens of *I. strobilina* and *I. felix* were collected from the forereef on the western (seaward) slope of Carrie Bow Cay. Specimens were collected of a sixth growth form with an irregularly massive body morphology (Sp. 1) from the *Rhizophora* prop roots of the Twin Cays and Blue Ground mangrove hammocks, and of a seventh growth form with an encrusting body morphology (Sp. 2) from the Blue Ground coral patch reef. Specimens of *I. strobilina* were also collected from the Blue Ground patch reef alongside Sp. 2. Each growth form was only found in one of three habitat types: coral patch reefs, seagrass beds, or mangrove prop roots, and were collected in close proximity to each other within a site; all Panamanian collections were made within an 8.1-km radius, the Floridan Ramose specimens shared a habitat with *I. campana* and are 13.1 km from Looe Key, and the Belizean sampling locations fall within a 7.5-km radius (Figure 1).

Sponge specimens were fixed in 90% EtOH, which was replaced at the 24-h and 48-h marks. 0.5 L seawater specimens were taken immediately adjacent to the sampled *Ircinia* in Panama and transported in opaque brown Nalgene bottles, subsequently concentrated via vacuum filtration through 0.2-μm Whatman filter papers, and then stored in RNA later. DNA extractions were made from the outermost 2 mm layer of the sponge tissues using DNeasy PowerSoil Kit (Qiagen, Hilden, Germany) and from the interior of the sponge tissue (<2 mm from the exterior pinacoderm) and the seawater filter papers using the Wizard Genomic DNA Purification Kit (Promega). 16S and 2bRAD data generation, as detailed below, were attempted for all sponge specimens unless otherwise stated and 16S data was generated for all seawater samples (Supplementary Table 1).

### Microbiome Analysis Using 16S Metabarcoding

To census the taxonomic microbial community compositions of sponges and seawater, we amplified the V4 region of the 16S rRNA (ribosomal subunit) from the DNeasy PowerSoil Kit DNA isolations using the primers 515f (5′ GTG YCA GCM GCC GCG GTA A 3′) and 806rB (5′ GGA CTA CNV GGG TWT CTA AT 3′) following the Earth Microbiome Project 16S protocol^1^. PCR reactions were conducted in 50 μL volumes with the following recipe: 25 μL of 2x HotStarTaq Master Mix, 1 μL of each primer at 10 μM concentration, 22 μL H_2_0, and 1 μL DNA template. Thermocycler conditions used an initial denaturing step of 95°C for 5 min followed by 35 of the following cycles: 94°C for 45 s, 50°C for 1 min, and 72°C for 1.5 min; and was completed with a final elongation step of 72°C for 10 min. The 16S sub-libraries were multiplexed using dual-indexing of 12-basepair Golay barcodes and pooled at equimolar concentrations following dsDNA quantification on a Qubit 3.0. The pooled 16S rRNA library was sequenced on a Illumina MiSeq in the lab of Dr. Noah Palm at Yale University using a V2 2 × 250 bp chemistry kit.

Quality filtering was performed on the 16S rRNA reads using the paired-end function in Trimmomatic v0.36 with the settings TRAILING:30 SLIDINGWINDOW:5:20 MINLEN:100 (Bolger et al., 2014). 16S rRNA reads passing initial quality filters were assembled into contigs, demultiplexed, and aligned to the V4 region of the SILVA v132 SSU reference sequence database in mothur v1.39.5 (Schloss et al., 2009). Following removal of chimeric sequences, OTUs were clustered at the 99% threshold using distance-based greedy clustering implemented in VSEARCH (Rognes et al., 2016). OTUs represented by only one or two reads were omitted from the final dataset to mitigate read error and contamination. Additionally, OTUs that were identified by SILVA as being from mitochondria, chloroplasts, or eukaryotes were removed from the dataset prior to downstream analyses. To infer which OTUs might be vertically transmitted, we used BLASTn to match the representative 16S rRNA sequences of our OTUs to a database of 16SrRNA sequences from bacteria that are putatively vertically transmitted in *I. felix* (Schmitt et al., 2007), downloaded from NCBI (Supplementary Table 2). OTUs were identified as being putatively vertically transmitted if they had 100% sequence identity over the entire length of the query.

Alpha diversity was calculated as the Shannon and inverse Simpson diversity indices using the R package vegan v2.5-6 (Oksanen et al., 2016), and the effect of source (host lineage and water) on these indices was inferred using Permutational Analysis of Variance (PERMANOVA), implemented in RVAideMemoire v0.9-79 using 10,000 permutations (Oksanen et al., 2016; Hervé, 2021). Beta diversity among the host species and differences in microbial community compositions between sponges and water were inferred with PERMANOVA based on Bray-Curtis dissimilarity using 10,000 permutations, implemented in vegan v2.5-6 (Anderson, 2001; Oksanen et al., 2016). Sponge microbiome compositions were visualized using a PCoA, and the overlap among the standard ellipse areas (SEAs) of each host species’ microbiome composition was calculated using SIBER (Jackson et al., 2011). We chose to use SEA, which encompasses 40% of the data and is analogous to a confidence ellipse in that it provides a way to compare community overlap in bivariate space, as it is robust to small sample sizes (Jackson et al., 2011). The number of unique and shared OTUs between seawater and sponge microbial communities was plotted with the eulerr R package^2^. Since seawater microbial communities were not sampled from Florida, analyses comparing sponge and seawater microbial communities were restricted to Panamanian and Belizean samples.

### Transcriptome Assembly, Post-assembly Decontamination, and Functional Annotation

RNA was extracted from the snap-frozen Ramose specimen by incubating a homogenized tissue fragment in Trizol and processing the resultant aqueous phase through the QIAGEN RNAeasy kit following the manufacturer’s instructions. The RNA extraction was sent to the Yale Center for Genome Analysis (YCGA) for library preparation via poly-A pulldown and sequencing on a Illumina NovaSeq 6000.

RNAseq reads were filtered and trimmed prior to assembly using the paired-end function of fastp v0.19.6 with the options –poly_g_min_len 10 -x –poly_x_min_len 10 (Chen et al., 2018). A first round of contaminant removal was performed on the RNAseq data using kraken v1.1.1 with the parameter setting –confidence 0.05 (Wood and Salzberg, 2014). To mitigate contamination of both eukaryotic and prokaryotic commensals, we supplemented the default kraken databases with custom databases built from a reference database of 356 metagenome-assembled genomes (MAGs) sourced from the same *Ircinia* host lineages studied here (Kelly et al., 2020) and a set of publicly available genomes downloaded from NCBI, which is predominantly crustacean and annelid as these taxa comprised the majority of eukaryotic commensals in the Ramose growth form (Supplementary Table 3). A second round of contaminant removal was performed using bbsplit.sh (Bushnell, 2014), implemented with a modification to default mapping parameters of maxindel = 2,00,000 and a reference database of the dereplicated *Ircinia* MAG dataset.

Reads passing the kraken and bbsplit.sh filtering steps were *de novo*-assembled using Trinity v2.8.5 (Grabherr et al., 2011). After assembly, a third round of contamination removal was performed using deconseq v0.4.3 with default parameters and the publicly available mouse, human, bacterial, archaeal, and viral deconseq reference databases (Schmieder and Edwards, 2011b). Functional annotations were then made for the assembly using the annotate function of dammit v1.0rc2^3^ including the UniRef90 annotations (–full) and the metazoan lineage-specific BUSCO group (Scott, 2016).

### *De novo* 2bRAD Assembly

To generate genome-wide SNP data for the host sponges, we constructed a 2bRAD (RADseq) library from the sponge DNA isolations produced using the Genomic DNA Purification Kit following the workflow of Wang et al. (2012), whereby all *Alf1* restriction sites were targeted for amplification with the primers 5ILL-NN (5′ CTA CAC GAC GCT CTT CCG ATC TNN 3′) and 3ILL-NN (5′ CAG ACG TGT GCT CTT CCG ATC TNN 3′) (Wang et al., 2012). The 2bRAD sub-libraries were dual-indexed with 12-basepair Golay barcodes and pooled at equimolar concentrations following dsDNA quantification on a Qubit 3.0. The pooled 2bRAD library was sequenced on a Illumina NovaSeq 6000 at YCGA.

The forward 2bRAD reads were trimmed to the 36-bp restriction fragments using the script 2bRAD_trim_launch.pl^4^, and quality filtered using cutadapt with the settings -q 15,15 -m 36 (Martin, 2011). Decontamination of the 2bRAD dataset (i.e., removal of reads of prokaryotic origin) was performed using bbsplit.sh (Bushnell, 2014) with default mapping parameters against the aforementioned dereplicated *Ircinia* MAG dataset. Following parameter optimization via the guidelines of Paris et al. (2017), we assembled the 2bRAD reads into loci *de novo* in Stacks v2.41 with the settings -m 3 -M 3 -n 4 (Catchen et al., 2013). Additionally, we filtered the data to require that a SNP be present in at least nine of the twelve of the populations (-p 9) and half of the individuals of a population (-r 0.50). For downstream population genetic analyses, we used only the first variant site from each 2bRAD locus to satisfy the assumption of independence among our SNPs.

### F_*ST*_*-*Outlier Detection Using 2bRAD Data and Annotation Against the Transcriptome

Using two methods, we detected *F*_*ST*_-outliers by treating each growth form and allopatric population of nominal species as a separate population. First, via BayeScan v2.1, a Bayesian software program that employs reversible-jump Monte Carlo Markov chains to estimate posterior distributions of *F*_*ST*_ values for loci under two alternative models, one with selection and another under neutral evolution (Foll and Gaggiotti, 2008). Loci with posterior *F*_*ST*_ values that deviate from expectations under the neutral model are identified as outliers. BayeScan was run for 5000 iterations with a thinning interval of 10 and a burn-in of 50,000. Outliers were identified from the output files and plotted using the R function plot_bayescan with a false discovery rate (FDR) of 0.10. Second, we identified outliers using the R package fsthet, an implementation of the *F*_*ST*_-heterozygosity approach of Beaumont and Nichols (1996) that identifies outlier loci against smoothed quantiles generated from the empirical SNP dataset (Flanagan and Jones, 2017). Using this approach, a given locus was identified as an outlier if it fell outside the 90% confidence interval (CI) of its heterozygosity bin.

To investigate the biological implications of selection in our dataset, we performed a two-step functional annotation. First, we mapped 2bRAD loci containing outliers by querying them against the assembled and annotated transcriptome using BLASTn with an *e*-value cutoff of 1e^–09^. Functional annotations of transcripts containing outlier loci were deemed reliable if the HMMER hits had *e*-values below 1e^–05^. Second, we queried outliers via BLASTx to the to the NCBI non-redundant (nr) protein sequences database.

### Species Delimitation, Species Tree Estimation, and Hybridization Using the 2bRAD Data

SNPs that were identified as *F_*ST*_-*outliers were removed from the SNP matrix prior to downstream population genetic analyses. Species delimitation was performed using Bayes factor delimitation with genomic data (BFD^∗^) (Leaché et al., 2014). 19 competing species grouping models were constructed based on plausible biological scenarios (Table 1). Each model was assigned an alpha = 1 and beta = 130 for the expected divergence prior θ and a prior distribution of gamma (2,200) for the birth rate prior λ^5^. Marginal likelihoods were then estimated for each competing model via path sampling analysis (Bouckaert et al., 2014), which was run for 50,000 generations with 28 path steps and a pre-burn-in of 25,000 generations. Bayes factors were calculated and compared following Kass and Raftery (1995). A species tree was estimated in SNAPP v1.3.0 for the best-supported species grouping model using four MCMC chains, each with a length of 1 million generations and a burn-in of 25%, totaling 3 million generations post-burn-in (Bryant et al., 2012). Likelihood estimates and trees were logged every 500 generations for the SNAPP species tree and path sampling analyses. Hybridization was inferred among the species identified by BFD^∗^ using STRUCTURE by setting the number of ancestral populations (*K*) to range from 3 to 12 and performing 10 runs for each *K* using an MCMC length of 200,000 and a burn-in of 50,000 generations (Evanno et al., 2005). Δ*K* was calculated to estimate the number of ancestral populations using the Evanno method implemented in Structure Harvester v0.6.94 (Evanno et al., 2005; Earl and vonHoldt, 2011).

**TABLE 1:** The species model representing each growth from and each population of nominal species as a distinct species (in bold letter face) received decisive support over competing models.

Species Model	Motivation	Number of Species	MLE	BF	Rank
**All growth forms separate species, split I. campana and *I. strobilina* by geography**	**Test population-level divergence.**	**12**	**−10712.77**	**2559.69**	**1**
Split *I. campana* and *I. strobilina* by geography, combine Massive A pink and Panamanian *I. campana*	Test if Massive A pink is a Panamanian I. campana phenotype	11	−10723.72	2537.80	2
All growth forms separate species, split *I. campana* by geography	Test population-level divergence.	11	−10743.48	2498.29	3
Split *I. campana* and *I. strobilina* by geography, combine Massive B and Panamanian *I. campana*	Test if Massive B is a Panamanian I. campana phenotype	11	−10743.96	2497.31	4
Split *I. campana* and *I. strobilina* by geography, combine Encrusting and Panamanian *I. campana*	Test if Encrusting is a Panamanian I. campana phenotype	11	−10745.95	2493.33	5
Split *I. campana* and *I. strobilina* by geography, combine Encrusting, Massive A pink, and Panamanian *I. campana*	Test if Encrusting and Massive A pink are Panamanian I. campana phenotypes	10	−10753.99	2477.27	6
Split *I. campana* and *I. strobilina* by geography, combine Massive A pink, Massive B, Encrusting, and Panamanian *I. campana*	Test if Massive B, Encrusting, and Massive A pink are Panamanian *I. campana* phenotypes	9	−10779.35	2426.54	7
Split *I. campana* and *I. strobilina* by geography, combine Sp. 2 and Belizean *I. strobilina*	Test if Sp. 2 is a Belizean *I. strobilina* phenotype	11	−10809.40	2366.44	8
All growth forms separate species, split *I. strobilina* by geography	Test population-level divergence.	11	−10895.09	2195.06	9
All growth forms separate species, combine *I. campana* and *I. stroblinia* populations	Full species model combining populations of nominal species.	10	−10924.28	2136.68	10
Combine massive A pink and Massive B	Both massive; shared geography.	9	−10940.90	2103.44	11
Combine Massive B and Massive A green	Shared habitat (STRI point); sympatry.	9	−10942.36	2100.53	12
Combine Massive A green and Encrusting	Sympatry	9	−10990.90	2003.45	13
Combines Sp. 1 and Massive A pink	Shared habitat type (mangrove)	9	−11064.83	1855.58	14
Combine Ramose and *I. campana*	Sympatry	9	−11073.93	1837.39	15
Combine Sp. 2 and Encrusting	Shared habitat type (coral patch reef)	9	−11224.58	1536.08	16
Combine Sp. 1 and Sp. 2	Sympatry	9	−11357.66	1269.93	17
Combine growth forms into one species	Are the growth forms different phenotypes of the same species?	4	−11826.39	332.46	18
Combine growth forms with *I. felix*	Are the growth forms different *I. felix* phenotypes?	3	−11992.62	−	19

### Test of Correlation Between Host Genetic Distance and Dissimilarity in Microbiome Composition

To test the hypothesis that genetic distance among the host *Ircinia* correlates with dissimilarity in microbiome composition, we performed Mantel tests implemented in the R package vegan v2.5-6 (Legendre and Legendre, 2012; Oksanen et al., 2016). A matrix of pairwise raw genetic distances was calculated from the SNP dataset with the *F*_*ST*_-outliers removed using the R package ape v5.4-1 (Paradis and Schliep, 2019), and a matrix of pairwise distances in microbiome compositions based on Bray-Curtis dissimilarities was constructed using vegan v2.5-6. The Mantel test was run for 10,000 permutations using the Spearman correlation method, although we also performed the test using Pearson and Kendall correlations to evaluate whether significance was sensitive to the correlation method used.

## Results

### Microbial Communities

Sequencing of 16S rRNA amplicons generated from the host *Ircinia* and seawater samples generated 8,777,283 paired-end raw reads, of which trimmomatic removed 67.64% (Bolger et al., 2014). Of the remaining 2,839,911 reads, 2,000,318 survived downstream quality control steps including removal of homopolymers and chimeric reads in mothur v1.39.5 (Schloss et al., 2009) (Supplementary Table 1). 392 OTUs were identified as chloroplasts and one OTU as mitochondrial. 11,042 prokaryotic OTUs were retrieved at the 99% clustering threshold, which SILVA v132 identified as belonging to 59 accepted and candidate phyla (Quast et al., 2013). The mean final read abundance per specimen was 15,127.09 ± 7458.258 (1 SD).

**FIGURE 2:**
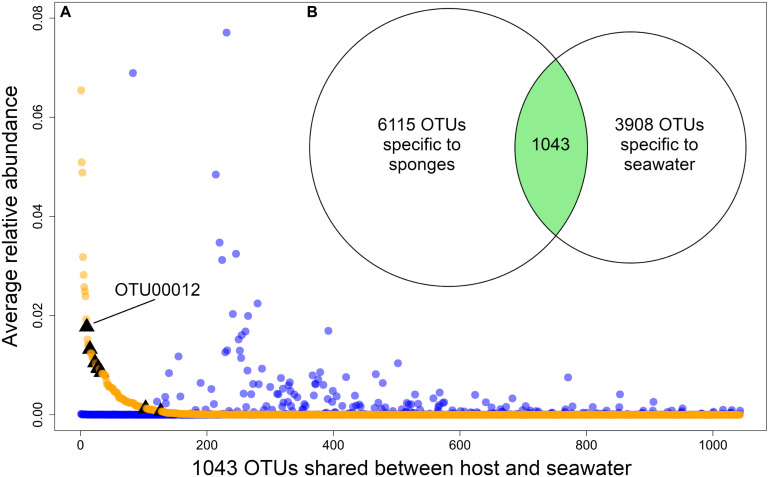
**(A)** Plot showing the relative abundances of the 1043 OTUs that are shared between sponges and seawater, restricted to Panama and Belize. Orange dots are relative abundances in sponges, blue dots are relative abundances in seawater. Black triangles mark OTUs that correspond to vertically transmitted bacteria in *I. felix* (Schmitt et al., 2007). **(B)** Venn diagram showing the number of sponge-specific OTUs, seawater-specific OTUs, and OTUs found in both sources. OTUs in the intersection of the two sources are plotted by relative abundance in **(A)**.

The source of each community had a significant (*p*-value < 0.05) effect on *H* and *1/D*, both when only sponge host lineages were considered (PERMANOVA for *H*: dof = 11, F = 8.30, *p*-value = 1e^–06^; PERMANOVA for *1/D*: dof = 11, F = 9.96, *p*-value = 1e^–06^) and when water sources were included (PERMANOVA for *H*: dof = 13, F = 11.35, *p*-value = 1e^–06^; PERMANOVA for *1/D*: dof = 13, F = 8.19, *p*-value = 1e^–06^) (Supplementary Figure 1). The microbiomes of *Ircinia* were compositionally distinct relative to seawater microbial communities (PERMANOVA: dof = 1, F = 58.61, *p*-value = 1e^–04^). *Ircinia* microbiomes were 1.45x as taxonomically rich at the OTU level relative to seawater microbial communities and contained 1.56x as many source-specific OTUs relative to seawater microbial communities. The two sources only overlapped by 1043 of the 11,042 OTUs, with 6115 OTUs only found in *Ircinia* and 3908 OTUs found only in seawater; however, the relative abundances of the OTUs found in both seawater and sponges were correlated with their source, where OTUs that were highly enriched in sponges were nearly absent in seawater, and vice versa (PERMANOVA: dof = 11, F = 6.54, *p*-value = 1e^–04^, Figure 2). The shared OTUs were also the most numerically dominant in the total dataset. When considering the fraction of OTUs found in the sponges, the shared OTUs had an average relative abundance of 8.59e^–04^ ± 4.04e^–03^ (1 SD), an order of magnitude greater than the average relative abundance of sponge-specific OTUs [1.71e^–05^ ± 1.25e^–04^ (1 SD)]. The same trend held for the seawater dataset, where the shared OTUs had a mean relative abundance of 8.3e^–04^ ± 4.42e^–03^ (1 SD), an order of magnitude greater than the average relative abundance of seawater-specific OTUs 3.35e^–05^ ± SD 1.28e^–04^ (1 SD). 8 OTUs were identical to 16S rRNA sequences from bacteria reported to be vertically transmitted in *I. felix*, all of which fell within the intersection of the sponge and seawater datasets, 6 of which were in appreciably higher relative abundances in sponges relative to seawater (Supplementary Table 4 and Figure 2).

**FIGURE 3:**
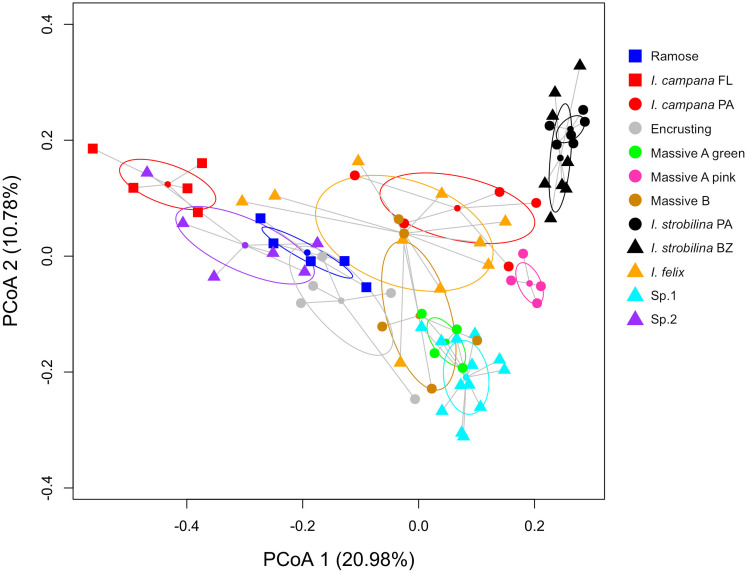
PCoA of microbiome compositions, normalized by relative abundance, for each host lineage. Ellipses are standard ellipse area (SEA). Squares are Floridian specimens (FL), circles are Panamanian specimens (PA), and triangles are Belizean specimens (BZ).

Each sponge lineage (growth form and population of nominal species) harbored unique microbiomes, evidenced by the significance (corrected *p*-value < 0.05) of 66 of 67 pairwise PERMANOVAs (Supplementary Table 5). Additionally, the microbiome compositions of each host lineage occupied distinct bivariate space on the PCoA, in which the standard ellipse areas (SEAs) of the host lineages had a mean overlap of 2.38 ± 6.11% (Figure 3 and Supplementary Figure 2). Within the nominal species *I. strobilina* and *I. campana*, geographically distant populations of conspecifics harbored significantly dissimilar microbiome compositions, although the distance between SEA centroids was 9.26x greater for the *I. campana* populations than the distance between the centroids of the *I. strobilina* populations (Supplementary Table 5 and Figure 3). 108 OTUs were found across all 10 host species and could thus be considered ‘core’ microbiota of Caribbean *Ircinia*. One OTU that blasted to the vertically transmitted symbiont 16S sequences was found across all 10 species, OTU00012, belonging to *Constrictibacter* (Proteobacteria, Alphaproteobacteria, Oceanospirillales, Oceanospirillales incertae sedis) (Yamada et al., 2011). Of the eight putatively vertically transmitted symbionts, OTU00012 had the highest average relative abundance across all host species (Figure 2).

### F_*ST*_-Outliers

A total of 118,948,690 single-end reads corresponding to *Alf1* restriction digests were generated using the 2bRAD pipeline, with an average of 1,383,124.30 reads ± 629,275.12 (1 SD) per specimen. After processing the reads with 2bRAD_trim_launch.pl^6^ and cutadapt (Martin, 2011), a total of 103,371,805 reads remained with an average of 1,201,997.73 ± 566,898.18 (1 SD) reads per specimen. Contamination screening against the MAGs of symbiotic prokaryotes via bbsplit.sh removed on average 74.52% ± 7.89% (1 SD) of reads per sample. Samples with less than 125,988.63 reads (1 SD below the post-decontamination per-sample mean) were omitted from further analysis. 66 samples remained with an average of 360,502.20 ± 180,813.27 (1 SD) reads per sample (Supplementary Table 1). Assembly of the 2bRAD data in STACKS (Catchen et al., 2013) requiring that a SNP be present in 75% of the populations and in half of the individuals per population produced 389 loci, 333 of which were variant.

51,117,656 2 × 100 bp RNA-seq read-pairs were generated. Decontamination of the transcriptomic data via kraken (Wood and Salzberg, 2014) removed 14.88% of reads and bbsplit removed a further 0.49%. After *de novo* assembly in Trinity (Grabherr et al., 2011), deconseq (Schmieder and Edwards, 2011a) removed 3791 sequences corresponding to 1.84% of the assembled transcripts. The final assembled transcriptome had a contig N50 of 1570 bps and a metazoan-specific BUSCO completeness score of 95.6%, 191,399 transcripts, 126,897 Trinity “genes,” and a GC content of 41.58%. 51,471 transcripts received functional annotations via HMMER, implemented in dammit (Scott, 2016), that met the *e*-value cutoff of <1e^–05^.

Fifty outlier loci were detected among the 333 variant 2bRAD loci; BayeScan identified 13, 10 of which were candidates for positive selection and 3 for balancing selection, and fsthet identified 43, 18 of which for candidates for positive selection and 25 for balancing selection. One locus identified as being under balancing selection and five outlier loci under positive selection were detected by both methods. 18 of these loci mapped to the transcriptome and passed the annotation criteria (Supplementary Table 6). Two of the positively selected outliers mapped to genes involved in cellular mechanics (FLNC and FLNB), and one to a gene involved in maintaining DNA integrity (BLM). One of the genes under balancing selection is involved in protein degradation (S8 Family Serine Peptidase), and five in host immune and stress responses (MAP3K, Rassf1, TES, PRSS21, and Kdm5b). One of the positively selected loci mapped to a mobile genetic element (TY3B-G) as did six loci under balancing selection (three POL, gag-pol, GIY-YIG, pro-pol-dUTPase). Additionally, the two genes annotated as TES and PRSS21 also contained viral recombination domains (phage integrase family); PRSS21 also contained a reverse transcriptase domain.

### Species Boundaries, Gene Flow, and Correlation Between Host Genetic Distance and Dissimilarity in Microbiome Composition

**FIGURE 4:**
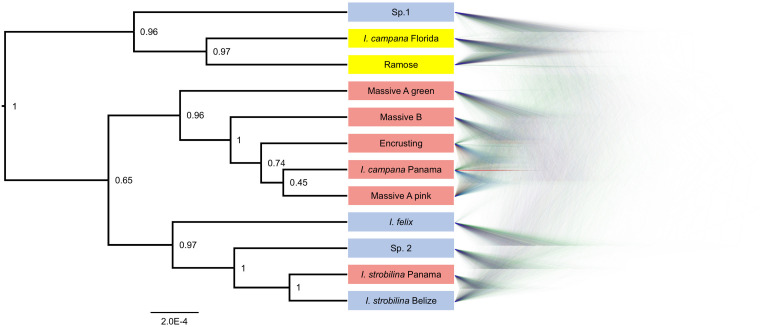
Phylogeny produced via SNAPP for the best-supported species grouping model in BFD^∗^. **Left**: consensus tree with posterior probabilities as node labels. **Right**: Densitree visualization of posterior tree distribution displaying most frequent topology in blue and alternative topologies in green and red. Tip labels are colored by geography: blue for Belize, yellow for Florida, and red for Panama.

BFD^∗^ lent highest support to the model representing each growth form and population of nominal species as a separate species (Table 1). The clades that were recovered via SNAPP showed a strong correlation with geography, especially with the Panamanian populations, which were monophyletic with the exception of *I. strobilina* (Figure 4). The Floridian and Panamanian *I. campana* were divided into two distinct clades that were connected by a deep node near the base of the tree; however, the *I. strobilina* populations were monophyletic.

**FIGURE 5:**
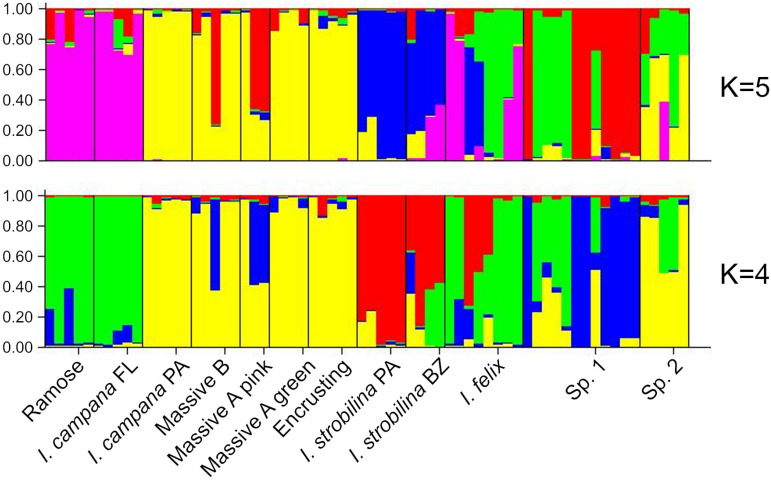
Structure plots of ancestries of neutrally evolving SNPs estimated for K = 4 and K = 5.

The best supported number of ancestral populations was identified unambiguously as K = 4, followed distantly by K = 5, by the Evanno method (Evanno et al., 2005) (Supplementary Figure 3). As the alleles are colored according to the ancestral population that they were predicted to derive from, both the K = 4 and K = 5 STRUCTURE plots showed patterns of hybridization indicative of interbreeding among *Ircinia* species, with higher rates of gene flow occurring within sites relative to across sites (Figure 5). In both plots, SNPs from both populations of *I. strobilina* were predominantly sourced from a shared ancestral population, whereas *I. campana* was split between two ancestral populations that coincided with geography.

Genetic distances among host *Ircinia* were significantly correlated with microbiome dissimilarities, independent of the correlation method used (Mantel statistic r (Spearman): 0.21, *p*-value = 1.4e^–03^; Mantel statistic r (Pearson): 0.21, *p*-value = 1.3e^–03^; Mantel statistic r (Kendall): 0.14, *p*-value = 9e^–04^).

## Discussion

Our results provide the first insight into the selective forces on specific genes that might underlie microbiome control and tolerance in high-microbial abundance (HMA) sponges. In particular, balancing selection was detected in immune system genes and positive selection in genes that concern the sponge’s mechanical control of its body and DNA repair. A heritable component of microbiome control might also be evidenced in our observations of dissimilarity between host microbiomes and seawater microbial communities. Combined with previous observations of vertical transmission of microbial symbionts in sponges (Enticknap et al., 2006; Schmitt et al., 2007; Sharp et al., 2007; Lee et al., 2009) and the fitness benefits to host sponges that can result from microbial farming (Weisz et al., 2007; Freeman and Thacker, 2011), our study provides a model that could help explain the persistence of host-microbial relationships throughout the evolutionary history of Porifera.

### Hidden Species Richness of Caribbean *Ircinia*

The growth forms of Caribbean *Ircinia* appear to be genetically distinct species, a hypothesis that held despite high rates of hybridization. Two notable features stand out on the species tree for the best-supported BFD^∗^ species model. The first is a strong correlation of the grouping of taxa with geography–all four Panamanian growth forms are monophyletic with the Panamanian population of *I. campana*, and the Floridian Ramose growth form is monophyletic with the Floridian population of *I. campana*. Second, and perhaps more striking, is the polyphyly of the *I. campana* populations. Conversely, the allopatric populations of *I. strobilina* are monophyletic on the tree.

The polyphyly of *I. campana* is a result that departs from conventional knowledge about this species, which is typically regarded as monophyletic and is one of the *Ircinia* in the Caribbean whose gross phenotypic features are expected to track species boundaries. Interestingly, a recent publication (Griffiths et al., 2021) used microsatellite genotyping of host sponges to uncover a trend of high genetic population structure among geographically distant *I. campana* populations, which were found to constitute four distinct admixture clusters. The results of Griffiths et al. (2021) and our current study are congruent in that Panamanian *I. campana* and Floridian *I. campana* appear in different clusters. Given their observations, Griffiths et al. (2021) suggested that *I. campana* might represent a case of cryptic speciation, although they restrict this proposal to a population inhabiting the Sapodilla Cayes that appeared particularly divergent from the remaining clusters. Given our BFD^∗^ results, we would like to append that cryptic speciation in *I. campana*, which curiously tracks microbiome divergence, could be a phenomenon that encompasses populations in addition to the one at Sapodilla Cayes.

Sympatric sister lineages on the phylogeny are separated by nodes as deep or deeper than the one connecting the allopatric *I. strobilina* populations (see the pairs of sister lineages: *I. campana* Florida and Ramose, and Massive A pink and *I. campana* Panama, Figure 4). We interpret the BFD^∗^ results, the depths of these nodes, and the distinct physical characteristics of each growth form as support for the hypothesis that each growth form represents a separate species, despite high rates of hybridization. We recommend against splitting *I. strobilina* into two species; we interpret the observed divergence among samples as population-level differences based on comparisons of the depth of the node joining the two allopatric *I. strobilina* populations to the depths of nodes joining sympatric species pairs. Taxonomic descriptions of each of these species are provided in a separate manuscript (Kelly and Thacker, 2020).

### Microbiome Compositions of Caribbean *Ircinia* Are Distinct From Seawater Microbial Communities and Are Unique Among Host Species

The most abundant microbes are those shared between host and seawater, whereas the OTUs specific to either sponges or seawater occur in relatively low abundances. None of these shared OTUs co-occurred in high relative abundances in both sources. Instead, sponges appear to be actively excluding some microbes that occur in high abundances in seawater and fostering high relative abundances of microbes that are only found in trace abundances in seawater. The presence of the majority of putatively vertically transmitted microbes in the set of microbes being maintained at high relative abundances in sponges supports this finding.

The microbiomes of each *Ircinia* host lineage are unique with regard to taxonomic composition, as the pairwise comparisons of the microbiome compositions of all allopatric species pairs and all but one sympatric species pair were significantly dissimilar. Combined with the distinctiveness of the compositions of sponge microbiomes relative to the seawater microbial communities and the correlation between genetic distances of the hosts and dissimilarities in microbiome compositions, a plausible explanation might be that a heritable mechanism in the sponges underlies microbiome assembly and potentially facilitates character displacement among sympatric species in addition to local adaptation to different habitats. If these microbes have an impact on the fitness of their hosts–which is a likely prospect given the high concentrations of photosynthetic pigments (Erwin and Thacker, 2007) and the presence of nitrogen metabolism (Archer et al., 2017) found in microbes inhabiting Caribbean *Ircinia*–these observations fit a model whereby microbiomes provide a conduit for ecological diversification within this genus.

The microbiomes of *Ircinia* species are also likely shaped on short-term ecological timescales to some degree; however, data describing the responses of microbiome compositions in Caribbean *Ircinia* to contrasting abiotic and biotic regimes across spatial and temporal scales are in short supply and were predominantly collected from *Ircinia* inhabiting other ocean basins. One study examining the Great Barrier Reef sponge, *I. ramosa*, found that microbiome compositions were stable when exposed to different salinity regimes (Glasl et al., 2018). Additionally, previous studies focusing on Mediterranean *Ircinia* spp. discovered that microbiome demographics are stable within a host species across seasons and throughout the range of their species distributions (Erwin et al., 2012; Pita et al., 2013). Recently, an analogous study was performed in the Caribbean that investigated patterns of beta diversity among the microbiomes of *I. campana* populations (Griffiths et al., 2019). This study discovered an effect of geography on the microbiome compositions of the populations (Griffiths et al., 2019), which is consistent with the significantly dissimilar microbiome compositions of the *I. campana* populations in the current study. To better understand how intersecting evolutionary and ecological forces dictate microbiome assembly in Caribbean *Ircinia*, we advocate for more studies performing long-term monitoring of microbiome compositions and reciprocal transplant experiments that could help elucidate how *Ircinia* are able to use their microbiomes to exploit unique resource pools in different habitats.

Recent work suggests that neutral processes might be influential drivers of microbiome assembly in sponges (Sieber et al., 2019). Furthermore, infidelity in the vertical transmission of sponge symbionts, such that only a subsample of the parents’ microbiome is inherited by offspring, could drive a distance-decay relationship in microbiome composition (Björk et al., 2019). Both of these processes could produce differences among the microbiomes of *Ircinia* species that are correlated with geography. For example, the repeated subsampling of microbiomes across host generations could magnify differences in microbiome community structure among host species, resulting in a pattern of ecological drift due to random chance. However, for these neutral processes to produce high beta diversity among host species, barriers to reproduction among host species must be strong enough to prevent hybridization, which would tend to homogenize microbiomes at local scales. These predictions are not consistent with our results from STRUCTURE analyses, because we found high rates of hybridization within sites (Figure 5) yet also found distinct microbiomes within sites (Supplementary Figure 2). An alternative model is that as the host’s immune system reaches maturity, *Ircinia* are able to select certain microbes to maintain at high abundances and are able to repress the abundances of others. This alternative model predicts a shift in microbiome composition throughout the lifecycle of the host, which has been previously reported in *I. felix* (Schmitt et al., 2007). Similar studies of symbiont transmission coupled with transcriptomic monitoring of the host’s immune system could help disentangle the drivers of beta diversity among these *Ircinia* species.

### The Role of Selection in *Ircinia* Evolution

The demands of living in a microbial world must be met by competent host defenses. Immune system genes are routinely subjects of balancing selection given the positive effect allelic diversity has in guarding against novel cellular assaults and in detecting a broader suite of foreign epitopes (Ferrer-Admetlla et al., 2008), and have been previously detected as being under balancing selection in sponges (Leiva et al., 2019). In our dataset, we detected multiple outliers under balancing selection involved in immune responses. The first two, MAP3K and Rassf1, are both expressed in the cytosol and are components of the Ras-Raf-MEK-ERK (MAPK/ERK) LPS-induced pathway that communicates molecular signals of a bacterial infection to drive the transcriptional changes necessary for an immune response (Orton et al., 2005). This signaling pathway also plays a role in cell-cycle mediation and in the induction of apoptosis, and is thought to be tumor-suppressing given the silencing of Rassf1 and somatic mutation of MAP3K in many cancers (Vos et al., 2000; Agathanggelou et al., 2005; Donninger et al., 2007; Stark et al., 2011). Two other oncogenes under balancing selection are TES (testisin) and PRSS21 (testisin precursor). Both are localized in the cell membrane. The former is a focal adhesion protein that controls cell proliferation (Coutts et al., 2003), the latter, a serine protease that is putatively involved in the regulation of proteolysis during germ line development (Manton et al., 2005). Our analysis detected both positive and balancing selection in biological processes involved in maintaining DNA integrity. Balancing selection was detected in Kdm5b, a histone demethylase expressed in the nucleus that signals double-stranded breaks in DNA (Li et al., 2014). Balancing selection was also detected in a member of the GIY-YIG nuclease family, which includes members that prevent the incorporation of exogenous DNA and are thought to preserve DNA integrity in basal metazoans (Dunin-Horkawicz et al., 2006; Brachner et al., 2012). Positive selection was detected in the BLM protein, which helps ensure accurate recombination during double-strand DNA break repair (Suzuki et al., 2016).

The concentration of bioactive compounds in *Ircinia* can be substantial, and some that have been identified as being produced by the symbiotic microbes are cytotoxic (Kalinovskaya et al., 1995; Prokof’eva et al., 1999; Hardoim and Costa, 2014). Many prokaryotic symbionts present in the adult sponges have also been observed in *Ircinia* larvae, where the toxic effects of the secondary metabolites are likely pronounced (Schmitt et al., 2007). Thus, balancing selection in immune system genes could be having a fitness effect in multiple cellular compartments (cell membrane, cytosol, and nucleus) and over the lifespan of the host. Additionally, the patterns of selection present in genes that promote DNA integrity, including oncogenes, could result in adaptations to the molecular apparatuses that prevent mutagenesis of host DNA in an environment that contains abundant foreign DNA and secondary metabolites.

Both balancing and positive selection were detected in viral recombination genes, providing the second account of selection in genes of a viral origin in sponge hosts (Leiva et al., 2019). Additionally, a recent study investigating the roles of phages in sponge symbionts (Jahn et al., 2019) discovered that bacteriophages of four Mediterranean sponge species contain genes that code for ankyrin repeat-rich proteins, which were previously demonstrated to aid symbionts in avoidance of phagocytosis by the host (Nguyen et al., 2013), and other genes that supplement the host bacterium’s metabolism. Since similar viral loci are under selection in the host, it may be the case that viruses are also transferring gene content to the sponges. Another possibility is that these genes are being erroneously detected as under selection, in that if they are in close proximity to other genes that are the true targets of selection, then our analysis could be detecting them as a result of a hitchhiking effect. However, this scenario does not preclude the possibility that the viral loci might still be involved in the mobility of the target of selection. Further research that can provide information on the physical relationship among these loci (i.e., using a reference genome) is required to better vet the potential for viruses to introduce adaptive genes to host sponges.

Two of the loci under positive selection (FLNC and FLNB) are involved in cellular mechanics, including the development and functioning of muscles in other metazoans (Leber et al., 2016; González-Morales et al., 2017; Mao and Nakamura, 2020); additionally, the copy of FLNC in our dataset contains a CH-like domain that is present in sperm flagella (Supplementary Table 6). These genes might be involved in contractions of the canal system and the mechanics of the flagellar beating of the choanocytes, which regulate the flow of water throughout the sponge (Leys et al., 2011). Some host organisms, such as legumes, control their microbiome by manipulating the microenvironment, which can deprive root nodules that are overgrown with cheater strains of rhizobia of oxygen (Kiers et al., 2003). Sponges might control their microbiomes analogously, as they are able to control which portions of their aquiferous canal system receive irrigation, resulting in a heterogenous distribution of oxygen that could impact physiologies of bacterial symbionts and thus change the microbiome composition (Hoffmann et al., 2008). Given that the growth forms are specific with regard to habitat preference, the divergence among these genes could further be compounded by the different hydrodynamic environments of coral reefs, seagrass beds, and mangroves (Guannel et al., 2016).

## Conclusion

Ecological divergence, as facilitated by the microbiomes of Caribbean *Ircinia*, could be enabled in part by the patterns of selection we detected in the genomes of the hosts including balancing selection at immunity genes and positive selection in genes involved in cellular mechanics and the maintenance of DNA integrity. Of special interest are the immunity genes as the innate immune system of sponges might play a central role governing host-microbial crosstalk and maintaining a healthy microbial homeostasis (Müller and Müller, 2003). Immunity pathways involving the mitogen-activated protein kinases (MAPKs) p38 protein kinase and c-*jun* N-terminal kinases/JNK are induced by LPS in the model sponge species *Suberites domuncula* (Böhm et al., 2001). One of the pathways detected as being under balancing selection here, Ras-Raf-MEK-ERK (MAPK/ERK), is stimulated by LPS in human cell lines and triggers downstream immune responses from the host (Sweet and Hume, 1996). Given the conservation of the actions of the other two MAPK pathways, a similar biological function could perhaps be performed by MAPK/ERK in *Ircinia*. Future work should test whether this pathway is inducible by microbes or microbial metabolites and investigate the implications of its role in the cell cycle for tolerance of the microbiome. By identifying the products of genes in this pathway and of other genes that we detected as being under selection, such research could further illuminate how sponges coexist with their microbiomes and how selection in host genomes drives microbially-mediated ecological diversification.

## Data Availability Statement

The datasets presented in this study can be found in online repositories. The name of the repository and accession number can be found below: National Center for Biotechnology Information (NCBI) BioProject, https://www.ncbi.nlm.nih.gov/bioproject/PRJNA700995.

## Author Contributions

JK conceived the study. JK, JL, DC, and RT designed the methodology and performed subsequent manuscript revisions. JK, JL, and TR performed data generation. JK and DC conducted formal analysis and investigation. JK and RT secured funding. JK wrote the original manuscript. RT supervised the study. All authors contributed to the article and approved the submitted version.

## Conflict of Interest

The authors declare that the research was conducted in the absence of any commercial or financial relationships that could be construed as a potential conflict of interest.

## Footnotes

1 https://earthmicrobiome.org/protocols-and-standards/16s/

2 https://github.com/jolars/eulerr

3 https://github.com/dib-lab/dammit

4 https://github.com/z0on/2bRAD_GATK

5 https://www.beast2.org/bfd/

6 https://github.com/z0on/2bRAD_denovo

## Supplementary Material

Supplementary Figure 1 Shannon (*H*) and inverse Simpson (*1/D*) diversity indices of *Ircinia* microbiomes and seawater microbial communities, visualized on the SNAPP species tree. Reported values are means with standard error of the mean.

Supplementary Figure 2 PCoAs inferred from the total sponge dataset with specimens and SEAs highlighted by geography emphasize divergence of microbiome compositions among host species within a site.

Supplementary Figure 3 Estimation of delta K using Structure Harvester. K = 4 ancestral populations of *Ircinia* received highest support via the Evanno method, followed by K = 5.
